# Hydrogen Peroxide as an Adjuvant Therapy for COVID-19: A Case Series of Patients and Caregivers in the Mexico City Metropolitan Area

**DOI:** 10.1155/2021/5592042

**Published:** 2021-07-03

**Authors:** Arturo Cervantes Trejo, Isaac D. Castañeda, Alejandra Cortés Rodríguez, Victor R. Andrade Carmona, M. del Pilar Calva Mercado, Liliana Salgado Vale, Montserrat Cruz, Sara Barrero Castillero, Lucero Chavez Consuelo, Mauricio Di Silvio

**Affiliations:** ^1^Carlos Peralta Professor and Chair of Public Health, Anahuac Institute of Public Health, Faculty of Health Sciences, Anahuac University Mexico, Naucalpan de Juárez 52786, Mexico; ^2^Carlos Peralta Chair of Public Health, Anahuac Institute of Public Health, Faculty of Health Sciences, Anahuac University Mexico, Naucalpan de Juárez 52786, Mexico; ^3^Bursary Scholar of Medicine, Anahuac Institute of Public Health, Faculty of Health Sciences, Anahuac University Mexico, Naucalpan de Juárez 52786, Mexico; ^4^Faculty of Bioethics and Faculty of Health Sciences, Anahuac University Mexico, Naucalpan de Juárez 52786, Mexico; ^5^School of Medicine, Universidad de Monterrey, San Pedro Garza García, Mexico; ^6^Faculty of Health Sciences, Anahuac University Mexico, Naucalpan de Juárez 52786, Mexico; ^7^Fellow of the American College of Surgeons (FACS), Medical Director of Hospital Mac Periferico Sur, Mexico City, Coyoacan 04700, Mexico; ^8^Faculty of Health Sciences, Anahuac University Mexico, Naucalpan de Juárez 52786, Mexico

## Abstract

Knowledge of the antiseptic effects of hydrogen peroxide (H_2_O_2_) dates back to the late 19th century, and its mechanisms of action has been amply described. Globally, many physicians have reported using H_2_O_2_ successfully, in different modalities, against COVID-19. Given its anti-infective and oxygenating properties, hydrogen peroxide may offer prophylactic and therapeutic applications for responding to the COVID-19 pandemic. We report a consecutive case series of twenty-three COVID-19 patients (of 36 initially enrolled) who had been diagnosed by their primary care physician (mean age: 39, range: 8 months–70 years; 74% male) and twenty-eight caregivers in the Mexico City Metropolitan Area who received a complementary and alternative medicine (CAM) telemedicine treatment with H_2_O_2_ taken by mouth (PO, at a concentration of 0.06%), oral rinse (mouthwash, 1.5%), and/or nebulization (0.2%). We describe the treatment program and report the response of the COVID-19 patients and their caregivers. The patients mainly recovered well, reporting feeling “completely better” at 9.5 days on average. Two (9%) were hospitalized prior to joining the study, and one did not fully recover. Patients frequently reported nausea and sometimes dizziness or vomiting related to the oral treatment. None of the twenty-eight caregivers in close contact with the patients reported contracting COVID-19. Given its low cost and medical potential and considering its relative safety if used properly, we suggest that randomized controlled trials should be conducted. These should include both SARS-CoV-2-positive and SARS-CoV-2-negative participants, with single or combined modes of administration of H_2_O_2_, to study the benefits of this simple molecule and offer safe guidance regarding its use by health professionals.

## 1. Introduction

A case series is described on twenty-three ambulatory patients diagnosed with COVID-19 (coronavirus disease 2019) and monitored by telemedicine, using an adjuvant therapy of hydrogen peroxide (H_2_O_2_), administered PO (per orem), by mouth rinses (oral gargles), and by inhalation routes. We report the clinical outcomes of the consecutive COVID-19 patients and their caregivers, who were treated by our medical team between May 11 and July 19, 2020.

Among other things, the team conducted a nonexhaustive review of the scientific literature to identify possible therapeutic and prophylactic alternatives. This review was supplemented by literature from the complementary, traditional, and integrative medicine fields. Our aim was to identify nonstandard therapeutic alternatives for treating viral infections, such as COVID-19, that could be easily and cheaply attained in Mexico over the counter and aid in the primary health response to the pandemic. With these requisites in mind, we identified this clinically useful molecule, hydrogen peroxide.

Dating back to 1888, Love et al. reported the use of hydrogen peroxide as an anti-infectious agent and described it as effective in treating numerous diseases including scarlet fever, diphtheria, runny nose, coryza, whooping cough, asthma, hay fever, and tonsillitis [1]. Specifically for viral diseases that attack the respiratory system, the first reported medical success using hydrogen peroxide therapy dates back exactly 100 years, when doctors Oliver and Murphy reported in The Lancet how they had successfully applied intravenous hydrogen peroxide to treat a group of patients with influenza; they reduced by half the mortality among this group of patients, which consisted of troops from the Indian army during the 1918-1919 Spanish Flu pandemic in the Mesopotamian valley [2].

Although the use of hydrogen peroxide therapeutically has generated great controversy in alternative medicine [3], this ubiquitous molecule is not just one of the many components that help regulate the amount of oxygen that reaches cells, but its presence is vital for a variety of other functions of the body. Many positive effects of hydrogen peroxide on the immune system response have been described, including the stimulation of monocytes and T-helper cells which help fight infections, the increased production of interferon-gamma, which has a role in immunoregulation, and the effect of decreasing the activity of B cells, which have a role in up-regulating the inflammatory response [4].

Known in medical terms as oxidative therapy or bio-oxidative therapy, hydrogen peroxide is a simple, well-studied, and useful molecule for a range of medical and sanitary applications. Hydrogen peroxide (H_2_O_2_) contains one more atom of oxygen than water (H_2_O) and is naturally produced in the human organism as a by-product of oxygen metabolism. It is metabolized by enzymes known as peroxidases and catalases, which decompose low concentrations of hydrogen peroxide into water and a free oxygen ion. “Hydrogen peroxide appears to be a ubiquitous molecule. We exhale it, excrete it and take it in from diet” [4]. It is produced endogenously for many functions of living organisms, and there is an abundance of scientific knowledge on this molecule, with sufficient documentation on its uses for sanitation, sterilization, and, importantly, diverse therapeutic modalities.

Given that the H_2_O_2_ molecule decomposes into water (H_2_O) and oxygen (O^−^), in appropriate doses, it is relatively safe for animal and human uses, as well as relatively nontoxic. In the late 1980s, Farr reported that hydrogen peroxide offers therapeutic benefits by directly destroying microorganisms through dual oxidative and oxygenating actions, caused by the released oxygen molecules [5, 6]. In more recent times, hydrogen peroxide has been widely hailed for use in the so-called “oxygenation therapy” in acquired immunodeficiency syndrome (AIDS), several types of cancer, heart and blood vessel diseases, immune disorders, infectious or pulmonary diseases, and many other ailments and conditions.

H_2_O_2_ has also been used in dentistry, alone or combined with other salts, since the start of the century [7, 8]. In a recent review, Marshall, Cancro, and Fischman have described scarce side effects on soft tissues after using 1%–1.5% H_2_O_2_ as a daily rinse, with over two years of follow-up. They report an in vitro study that found that 3% H_2_O_2_ effectively inactivated many virus types, discovering that “coronaviruses and influenza viruses were the most sensitive” [8]. They further state that “since SARS-CoV-2 is vulnerable to oxidation, preprocedural mouth rinses containing oxidative agents such as 1% H_2_O_2_ have been suggested to reduce the salivary viral load” [8].

Brownstein et al. from Wayne State University School of Medicine, who has been applying oxidative therapies for over two decades, recently reported a novel treatment program combining nutritional and oxidative therapies against COVID-19. They used hydrogen peroxide to successfully treat the signs and symptoms of patients diagnosed with COVID-19 [9]. They base their treatments on a combination of oral, intravenous, intramuscular, and nebulized hydrogen peroxide. Their approach has resulted in zero deaths and the recovery of 107 COVID-19 patients [9].

The Food and Drug Administration (FDA) and other official health agencies have also approved the use of hydrogen peroxide as a disinfectant for medical equipment and facilities that have been in contact with the SARS-CoV-2 [10]. In vitro studies demonstrate the efficacy of H_2_O_2_ in the vapor phase as a viricide against pathogenic viruses such as SARS-CoV and MERS-CoV [11]. Other studies, regarding viral inactivation in surfaces using diverse disinfectants, including hydrogen peroxide, have also been reported [12, 13].

Recently, the medical hypothesis that hydrogen peroxide is effective against the COVID-19 coronavirus, as well as other viral pathogens and bacteria, has been further advanced [14, 15]. Caruso et al. on the front lines against the COVID-19 outbreaks in Naples, Italy, recommend the need for clinical protocols and research on oxidative therapies regarding COVID-19 [14]. After review of the literature, they propose that the application of hydrogen peroxide to the epithelial cells of the nose, mouth, and throat could well be “extremely effective” against viruses, including SARS-CoV-2 [15]. Gansky's clinical trial at UCSF titled “Effect of antiseptic mouthwash/gargling solutions and pre-procedural rinse on SARS-CoV-2 load” is another example of current research on the subject [16].

The Italian group further proposes that a COVID-19 disinfection regimen by gargling with hydrogen peroxide through mouth rinses (and gargles), two to three times a day, could be useful for the disinfection of COVID-19 from the oral cavity. They also recommended nasal washes with a peroxide nebulizer two times a day. In their opinion, “the effectiveness of hydrogen peroxide-based therapeutic regimen would be verifiable by a significant reduction in the rate of hospitalizations and respiratory complications in patients positive to SARS-CoV-2” [14, 15]. Gansky's clinical trial at UCSF titled “Effect of antiseptic mouthwash/gargling solutions and pre-procedural rinse on SARS-CoV-2 load” is another example of current research on the subject [32].

A further example is Khan et al.'s clinical trial of gargling agents in reducing intraoral viral load among COVID-19 patients based in Pakistan [17]. This quadruple blind trial provides useful information because it is randomized and controlled, and one of their treatments is a 1% hydrogen peroxide gargle. The study design, which includes patients in parallel groups, using diverse types of gargles and nasal lavages, has as outcome measure the intraoral viral load of SARS-CoV-2, and their results will test the hypothesis of H_2_O_2_'s usefulness for handling the current pandemic, especially in overburdened areas currently suffering consecutive waves of COVID-19 transmission. 

Given the public health emergency facing Mexico and what ethics mandate from us as physicians, being aware of the increased demand expected for healthcare services in Mexico [18], as well as the absence of effective and approved therapeutic regimens, we hypothesized that hydrogen peroxide, an antiseptic agent, could play a pivotal role in reducing the severity and duration of the illness in patients and also preventing transmission among caregivers and close contacts. We therefore provided ambulatory treatment using hydrogen peroxide as a complementary therapeutic alternative for twenty-three COVID-19 patients. This treatment was extended in a prophylactic modality to twenty-eight caregivers or persons in close contact with the patients.

Here, we present a case series with our findings and provide some insight and recommendations, which could be useful to the scientific community as an adjuvant treatment during the pandemic's evolution as it affects different countries.

## 2. Materials and Methods

This was a single-center case series. All patient consultations were provided in an ambulatory care setting by telemedicine, using traditional telephone calls and WhatsApp messaging. Medical consultations were provided by licensed physicians from the Faculty of Health Sciences, Anahuac University Mexico, along with five recent medical graduates, and from a private hospital in Mexico City.

Inclusion criteria were symptomatic patients having a COVID-19 diagnosis made by their primary care physician, with a prescribed medical treatment; positive RT-PCR (reverse transcriptase polymerase chain reaction), computed tomography (CT) scan, or chest X-rays suggestive of COVID-19; having access to a healthcare service provider; consent to participate in telemedicine service; agreement on daily follow-up calls; providing informed consent for the ambulatory telemedical management of their condition with the use of hydrogen peroxide as a complementary experimental alternative; and not being in a serious stage of the illness, nor presence of symptoms of severe respiratory distress that requires hospitalization.

Of the initial 36 patients enrolled, two cases were excluded due to loss of follow-up, five were excluded due to nonacceptance of the adjuvant treatment with hydrogen peroxide, one discontinued the treatment, three did not provide test results, and one was misdiagnosed with COVID-19. This meant that only 23 of the initial patients fit the inclusion criteria for this case series report.

Information collected included age, sex, date of birth, initial date of symptoms, care provider, medical history, medications, and supplements taken. The number of days of illness before being recruited into this treatment program was also documented. For chest X-ray images and CT scans, we used the reports provided by radiologists and validated the images ourselves. SARS-CoV-2 testing was done by a professional using RT-PCR by independent laboratories and nasal swab sampling.

A concentrated solution of 30 mL of tridistilled or ultrapure hydrogen peroxide at a concentration of 35% (115 volumes) was provided to each patient and each caregiver in 30 ml droppers. Given the risk of misuse because of the oxidizing corrosive nature of the concentrated H_2_O_2_ solution, precise instructions on preparing the dilutions were given both verbally and in writing (described in Table 1). Purified bottled water or simple tap water was used to dilute the H_2_O_2_. Patients and caregivers were required to purchase a commercially available nebulizer for respiratory treatment. Clinical monitors accompanied caregivers and patients via phone calls during the opening of the packaging and accompanied the preparation process to ensure the adequate knowledge and use of the solution and preparation for each different administration route.

Patient and caregiver interventions included a basic training for measurement of temperature, oxygenation, respiratory frequency, and cardiac rate and keeping a record of clinical signs and symptoms. Both the oral and nebulized administration were given depending on each patient's tolerance. We made a note of adverse effects such as nausea, pharyngeal or nasal irritation, and vomiting. As the symptoms improved, the oral intake and the nebulizing could be reduced by the patient, coordinated by the clinical monitors and attending physicians. Whenever patients presented severe clinical deterioration (oxygen saturation <85%, dyspnea, tachycardia, extreme fatigue), hydrogen peroxide was suspended, and the subjects were instructed to seek medical attention in a hospital. Adverse effects were monitored in accordance with guidelines issued by the Mexican Sanitary Authority (COFEPRIS) and Mexican Official Norm of Pharmacovigilance (NOM-220-SSA1-2016).

Disease progression was evaluated based on clinical criteria: first *improvement* (or feeling of improvement endpoint) was defined as the positive change that each of the patients report to the clinical monitor during the daily follow-up interview. Clinical monitors were trained to recognize this positive change when the patient, during the interview, in addition to spontaneously reporting a feeling of improvement, reported a decrease in the following symptoms: headache, asthenia/adynamia, general discomfort, and dyspnea/shortness of breath. *Completely better* or clinical remission of symptoms endpoint was defined as the total or almost total absence of any of the following symptoms during the daily follow-up interview for each case: headache, asthenia/adynamia, general discomfort, and dyspnea/shortness of breath. This second endpoint entails the clinical remission of symptoms and therefore allows the clinical monitor to declare the end of the acute period of the disease.

Antipyretics and nonsteroidal anti-inflammatory drugs were used as each clinical case required. Concomitant medications for comorbidities were also continued as managed by their usual healthcare providers. Additional medications used by patients included antiretrovirals, antibiotics, and in two cases hydroxychloroquine.

## 3. Results

The baseline characteristics of the patients are described in Table 2. The age ranged from 8 months to 70 years with a mean age of 39 years. Six patients were female (26%) and 17 male (74%). Three patients were active smokers and two were passive smokers. Seven (30%) patients were overweight and two (9%) were obese according to body mass index (BMI); body measurements were not available for the rest of the patients. The major comorbidities included systemic arterial hypertension (22%), diabetes mellitus (17%), and gastroesophageal reflux (17%).

The clinical symptoms are illustrated in Table 3. The most common symptoms were cough, headaches, and weakness (asthenia/adynamia).

As seen in Table 4, among the confirmed COVID-19 patients, the most common symptoms were also cough, headache, and asthenic/adynamic feeling. Fever was present in 4 patients (36%) while pneumonia was diagnosed in 2 patients.

Table 5 presents the diagnostic and imaging studies performed on the 23 patients and a summary of the disease course for all of them. Twelve patients were tested for COVID-19 with RT-PCR and 92% were positive. Fourteen had imaging (chest X-ray or CT scan) studies. For comparison purposes, the symptomatology of the whole group of patients will be presented separately from symptoms in the laboratory-confirmed COVID-19 cases. Most patients did not have complementary laboratory studies (it is important to know that over half of these patients had limited resources). Two patients presented deteriorating conditions and were hospitalized. None of the 23 patients died.

On average, most patients felt the first improvements within the first two and a half days since starting the experimental H_2_O_2_ treatment. Patients were “mostly better” at an average of 6.2 days, and patients were “completely better” in an average of 9.5 days. Symptomatology was mostly better within 2 to 11 days and completely better from 3 to 15 days for the most part.

Table 6 presents the hydrogen peroxide treatment modality received by patients and lists concomitant classes of medications that were administered during the course of the disease. Most patients received a full spectrum of pharmacologic support with antimicrobials, analgesics, and antipyretics. Seven patients received antivirals and two patients were reported to receive hydroxychloroquine.

**Figure 1:**
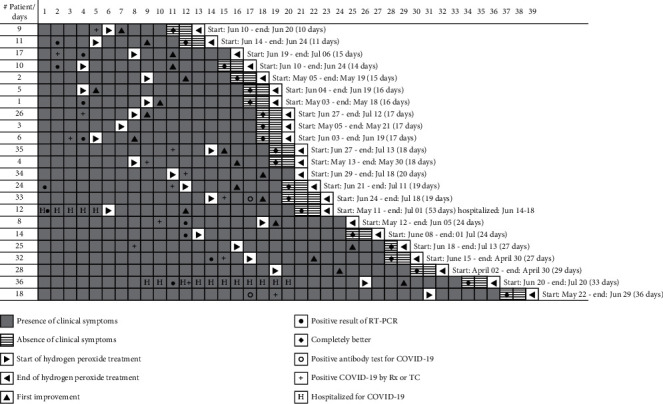
Timeline and disease course of twenty-three consecutive COVID-19 patients with CAM management.

Figure 1 illustrates the disease course for the twenty-three consecutive patients, ranked by length of the SARS-CoV-2 disease duration. The vertical axis is the patient number, and the horizontal axis represents the days since clinical onset of the disease. Patients are ordered by duration of disease and not by consecutive appearance. Patient #1 was the first to enter the case series on May 1^st^, and patient 36 was the last to enter the series on June 20^th^. Patient #36 was the last to exit the series on July 20^th^. Two patients entered the treatment protocol after being released from the hospital for COVID-19 disease. Patient #12 had been hospitalized for 5 days and, after being stabilized, was sent home to continue as outpatient when he entered the study. Patient #36 had also been stabilized after 12-day hospitalization, but his symptoms continued for five days after discharge. He was accepted for treatment and reported a first improvement on the third day and complete recovery five days later.

The graphic displays in shades of gray the presence or absence of clinical symptoms as well as the start and end of the hydrogen peroxide treatment. It also illustrates additional clinical events of relevance. Key milestones for the evolution of each case are also presented and include presence or absence of clinical symptoms (gray shading), start and end of hydrogen peroxide treatment (arrows), day of first improvement (triangle) and day of feeling “completely better” (circle), hospitalization days (letter H), confirmatory RT-PCR exam (dot), positive serum antibody exam (diamond), and confirmatory CT scan or X-ray (plus sign).

Table 7 presents a summary of adverse effects from the use of hydrogen peroxide. It includes details regarding the gender and age of the patients, the date and time of the effect, and the route of administration, also, the type and severity of the adverse effect, possible causes, if it represented a security problem, countermeasures taken, and consequences or after-effects. Eight patients reported twelve episodes of mild adverse effects, half from the oral and half from the nebulized routes of administration. Out of six adverse effect reports from oral administration, five were possibly not related to the use of H_2_O_2_. These patients were similar in that they reported gastroesophageal symptoms prior to administration or were using several PO medications simultaneously, without gastric protection. Out of the six adverse effect reports from the nebulized route of administration, four were possibly not related to the use of H_2_O_2_ and two were due to accidental misuse. All adverse effects had complete recovery, with no after-effects.

Of the 28 caregivers who received instructions for prophylactic mouth rinsing and gargles with H_2_O_2_, none reported acquiring the disease at the closing of the study or after the 30-day follow-up period. Three caregivers (11%) reported minor safety issues with the handling of the H_2_O_2_ concentrated solution. On two occasions, small amounts of the solution were spilled during preparation and reached the skin surface of hands or fingers of the person preparing the dilution. This caused immediate burning sensation and whitening of skin surface which lasted between 20 minutes and 30 minutes. Treatment was indicated as washing hands and rinsing in cold water. In one case, an undiluted drop of H_2_O_2_ accidentally remained on edge of the glass used for gargling. A canker sore in the person's gum occurred as undiluted H_2_O_2_ touched the oral mucosa. All these cases resolved favorably, without complications or after-effects.

Figure 1 illustrates the start of H_2_O_2_ therapy (forward arrow) and the day of first improvement (triangle). In ten patients, “first improvement” was reported on the first day of treatment with hydrogen peroxide. In many of these cases, the improvements were noted since the first applications of the hydrogen peroxide. On average, the duration of disease starting from the application of hydrogen peroxide to recovery was of 8 days. The minimum days for complete recovery were 4 and the maximum were 14. All patients except number 17 had a complete recovery. Two patients were hospitalized and discharged prior to inclusion in the study.

## 4. Discussion

“When the river sounds, it means it's carrying water” [19]. We have described the use of three concomitant treatment modalities with hydrogen peroxide (mouth rinse and gargles, oral, and respiratory) which have proven to be safe and well tolerated among a group of 23 consecutive COVID-19 patients. Complementary and alternative medical treatments such as this, using hydrogen peroxide, may have played a significant role in the rapidly improving clinical characteristics and health outcomes observed among our consecutive twenty-three COVID-19 patients, and thus, it deserves further investigation. The age of the patients at a mean of 39 was relatively young compared to those who experience severe COVID-19, and without a control, it cannot be concluded that the treatment contributed to reducing the duration or severity, considering the natural history of disease [20, 21].

Overall, most patients had a disease that lasted between 15 and 30 days, and only three patients had a disease that lasted more than 31 days. The shortest duration was 11 days in patient #9. In four patients, the duration was 14 days or less. Patient #12 came to us after being in the hospital for 5 days and was admitted considering his first day when he received the positive result of the RT-PCR result; the duration of his disease was 53 days. The start of his disease was much longer than is reflected in Figure 1.

For over four decades now, proponents of oral therapies with hydrogen peroxide have existed in the CAM and integrative medicine circles. They have argued in favor of the therapeutic effects of this molecule for multiple human ailments, ranging from cancer to diabetes, and spanning diseases of the cardiovascular, respiratory, gastrointestinal, and immune bodily systems [5, 6, 22–26].

Yoon et al. have shown that the SARS-CoV-2 viral load is consistently high in the saliva, higher than that in the oropharynx during the early stage of COVID-19 [27]. Their finding suggests that SARS-CoV-2 might be secreted from the salivary glands. Therefore, mouth rinsing with an antiseptic agent could be effective in reducing the SARS-CoV-2 viral load in the saliva and controlling droplet transmission for a short-term period. The hypothesis that oral gargle agents and nasal lavages with hydrogen peroxide dilutions could reduce the oral and the nasopharyngeal viral load and could help improve the immune response to COVID-19 and its symptoms seems to be *well founded* and is supported by our observations in this case series report [14, 15].

Our enteral supplementation of hydrogen peroxide at 0.06% was based on the twofold assumption that (1) the SARS-CoV-2 attacks the gastrointestinal system of many patients and (2) the gastrointestinal system may become a modulator for circulating oxygen in the body. It is known that the gastrointestinal tract is about 40% more efficient at assimilating oxygen than the lungs; thus, the oral administration of hydrogen peroxide is a very effective way of getting therapeutic oxygen into the body [25].

In the oral administration, a frequent complaint was that it caused nausea, sometimes dizziness, and vomiting and was not easily tolerated. Incremental dosing was instructed; with gradual increments, tolerance and acceptance of enteral administration were achieved.

Successful nebulization with oxidizing solutions for the symptomatic treatment of airway infections has also been recently reported for COVID-19 cases [23]. Most of our patients reported immediate relief of respiratory symptoms and documented improved oxygenation as measured by their pulse oximeter with the nebulization. In addition to the reduction in the duration (compared to clinical progress and outcomes for Mexican patients), we observed a possible reduction in the severity of the disease and a perceived reduction in symptoms by most patients.

Seven out of the twenty-three patients were responsible for their own care. Of the other sixteen patients, a total of twenty-eight caregivers or people in close contact with the COVID-19 patients (living within the same household) self-administered prophylactic hydrogen peroxide mouth rinse and gargle recommendations, as described within the methodology. At follow-up, one month after the disease had receded, none of the caregivers who used prophylactic mouth rinsing and gargles reported acquiring the disease.

Because we gave the patients/caregivers a 35% solution of H_2_O_2_ to dilute themselves, there was a risk of severe harms by accidental spillage or inhalation of the concentrated solution or by accidental misdilution. These safety issues were emphasized with patients and caregivers, and specific instructions were added to keep away from children. Ideally, only prediluted solutions should be dispensed to patients to avoid possible harm from misuse of a highly oxidizing corrosive solution.

The systematic review of Ortega et al. [12] to detect studies that document the virucidal effect of hydrogen peroxide concludes that there is no evidence specifically for its use through mouthwashes, which is understandable, since documenting the virucidal effect of rinses had not been the great interest of the scientific community, as Ortega himself makes clear. There is no study in the literature demonstrating the efficacy of H_2_O_2_ as a virucidal agent for surface disinfection either. During their review, they found only one in vivo study that evaluated the efficacy of a product (Listerine) in reducing HSV-1 in the saliva of patients with active lip lesions [28]. The lack of a standardized method to demonstrate how to verify the virucidal effect in nonstandardized samples from the nasopharyngeal and oral cavity becomes a challenge to develop clinical research studies aimed at generating evidence of the virucidal effect of any mouthwash.

This can be confirmed in the systematic review carried out by Cavalcante-Leão et al., aimed at detecting evidence on the effectiveness of mouthwashes in reducing viral load in COVID-19. They recommend further research (mainly randomized clinical trials), given the scant evidence found [29]. On the other hand, efforts such as that of Gottsauner and collaborators [30], to question the effect of hydrogen peroxide at 1% on intraoral viral load in 10 subjects positive for SARS-CoV-2, are valuable to warn the scientific community about the importance of not advancing clinical measures and recommendations for the application of hydrogen peroxide without the support of scientific studies that present conclusive data.

Currently, the clinical trials database of the National Library of Medicine contains 15 registered trials about the use of hydrogen peroxide as a prophylactic mouthwash, in concentrations ranging from 1 to 3%. This fact highlights the interest of the global scientific community in the use of this substance to combat the SARS-CoV-2. However, there are no registered trials for use of H_2_O_2_ with oral or nebulized applications.

Amidst the staggering toll of the pandemic, there has been scarce scientific interest in complementary and alternative medical treatment modalities against COVID-19, such as this one using H_2_O_2_, which we believe merits further scientific scrutiny. Given the possible therapeutic and prophylactic value that has been observed in this small number of patients, caregivers, and close contacts, we believe that the molecule merits further scientific scrutiny. We considered that hydrogen peroxide could be easily distributed at mass scale, could help slow the transmission among healthcare professionals and vulnerable populations, and could even act as a prophylactic agent.

This is the reason why we release this case series report, with the hope of raising interesting questions in the medical field and stimulating discussion as well as much needed clinical research on the subject.

## 5. Limitations

The results obtained are only observational and not generalizable to the entire population and therefore do not prove causation. Another limitation is selection bias, given that patients were not selected randomly and were accepted consecutively as they approached us and fulfilled our inclusion criteria. Another limitation is that the pharmacological treatment of all patients in the case series was not standardized, and during the study implementation primary care physicians prescribed medication such as hydroxychloroquine and ivermectin, now known to have no medical improvement in the disease. This makes it difficult to fully attribute hydrogen peroxide's capacity to improve the disease. We also did not provide case controls. Given the public health emergency, patients were accepted rapidly and had diverse comorbidities and concomitant medical treatments that could interfere with the treatment and influence the health outcomes.

Thus, further study is needed with a standardized protocol of medical treatment (unavailable now) given the novelty of the disease we are assessing and the lack of resources for implementing a double-blind randomized clinical trial.

As with any report aiming to expose the benefits of telemedicine, a limitation to this method of care that must be addressed is the difficulty in gauging whether the caregivers or patients are correctly administrating the therapies prescribed or being rigorous in the vital sign recording, our primary measurement for improvement. It is most definitely a trust exercise, but one that is worth the risk based on our findings.

A minor, yet worth addressing, limitation is that, due to the ambulatory care given to our patients, paraclinical studies were not readily available, and only the patients with more economic capacity were able to acquire paraclinical posttreatment. This is primarily the reason why our most reliable improvement metrics were signs and symptomatology.

In the official guidelines for the treatment and management of COVID-19 ambulatory patients, published in February 2020 and updated in July 2020 by the Mexican Ministry of Health, the indication for treatment for COVID-19 patients is merely symptomatic [31]. This means that patients are sent home with acetaminophen as the only treatment. There are no pharmacological alternatives presented as substitutes for Mexican practicing physicians, rendering the management of the disease a complicated challenge for many. Complementary and alternative medicine has a lot to offer to fill this inhumane void.

## 6. Conclusions

Hydrogen peroxide is a widely used, highly accessible, and available chemical compound whose efficacy has been demonstrated on several human viruses, including coronavirus and influenza viruses [32]. It is possible that hydrogen peroxide, by diverse routes of administration and mechanisms of action, could exhibit a therapeutic and/or prophylactic effect against SARS-CoV-2.

- The concentrations of H_2_O_2_ that we used for mouth rinses, for enteral administration, and for nebulized application are safe, as no serious side effects were reported in either of the three modalities of administration.
- Research is needed to determine the full potential of complementary and alternative therapies such as those with hydrogen peroxide, for use in prophylaxis and treatment against COVID-19.
- We strongly encourage the rapid development of randomized controlled trials to study the benefits of oral (enteral), mouth and nasal rinse, and vaporized applications of hydrogen peroxide against SARS-CoV-2, in singular use or therapeutic combinations.
- Although further clinical studies are required to evaluate the safety and efficacy of antiseptic mouthwashes against SARS-CoV-2, the prophylactic application represents a promise for widespread uses among the general population, especially the vulnerable and highly exposed groups.

## Figures and Tables

**Table 1:** Patient and caregiver instructions per administration route.

Route	Frequency	Duration	Other indications
Per orem (0.06% H_2_O_2_) (0.2 vol.)	Every 8 hours	16 days	Diluted in bottled or tap water, as tolerated
Nebulized (0.2% H_2_O_2_) (0.7 vol.)	Every 4 to 8 hours for 5 to 15 minutes as tolerated, or hourly until improvement	16 days	Diluted in bottled or tap water. Nebulized (not vaporized) in containers from 10 to 30 mL with cold nebulization devices
Prophylactic mouth rinsing and gargles (1.5% H_2_O_2_) (4.95 vol.)	Gargle for 30 seconds in oral cavity and 30 seconds in the back of the throat, every 8 to 12 hours	Daily	Diluted in clean tap water

**Table 2:** Case series with H_2_O_2_ management: characteristics of the patients.

Patient characteristics	Number	Percent
Total patients	23	100

Age		
Range	8 months–70 years	
Average age	39	
Median age	39	

Gender		
No. of males	17	74
No. of females	6	26

Comorbid conditions		
Never smoked	14	61
Normal weight	11	48
Overweight	7	30
Hypertension	5	22
Ceased smokers	4	17
Diabetes	4	17
Gastroesophageal reflux disease/gastropathy	4	17
Active smokers	3	13
Passive smokers	2	9
Obese	2	9
Hypothyroidism	1	4
Cancer	1	4
Hyperuricemia	1	4

**Table 3:** Symptoms for total sample of patients (*n* = 23).

Symptoms	Number	Percent
Cough	20	87
Headaches	19	83
Asthenia, adynamia	19	83
Malaise	15	65
Myalgia or arthralgia	14	61
Chills	13	57
Fatigue	11	48
Gastrointestinal symptoms (diarrhea, vomiting, anorexia, hematochezia, loose stools, pain)	10	43
Dyspnea	10	43
Fever	8	35
Odynophagia	6	26
Pneumonia	6	26
Anosmia	5	22
Upper respiratory infections	4	17
Dizziness	2	9
Conjunctival inflammation (eye redness)	2	9
Palpitations	1	4

**Table 4:** Symptoms for laboratory-confirmed COVID-19 patients (*n* = 11).

Symptoms among COVID-19 positive cohort	Number	Percent
Headache	9	82
Cough	9	82
Asthenia, adynamia	9	82
Myalgia or arthralgia	8	73
Malaise	8	73
Chills	7	64
Fatigue	7	64
Fever	4	36
Upper respiratory infections	2	18
Gastrointestinal symptoms (diarrhea, vomiting, anorexia, hematochezia, loose stools, pain)	2	18
Dyspnea shortness of breath	2	18
Pneumonia	2	18
Odynophagia	1	9
Conjunctival inflammation (eye redness)	1	9

**Table 5:** Clinical course, laboratory, and imaging studies (*n* = 23).

Studies performed and disease course	Number	Percent
Tested for COVID-19 (labs only)	12	52
Tested positive for COVID-19 (RT-PCR)	11	92
With chest X-ray or CT scan	14	61
Without chest X-ray or CT scan	9	39
With laboratory studies	3	13
Without laboratory studies	20	87
Hospitalized	2	9
Death	0	0
	Days	
First improvement average days (min–max)	2.5 (1–8)	
Mostly better average days (min–max)	6.2 (2–11)	
Completely better average days (min–max)	9.5 (3–15)	

**Table 6:** H_2_O_2_ and conventional pharmacological treatments used by patients.

Patient interventions	Number	Percent
Oral H_2_O_2_	22	96
Nebulized H_2_O_2_	17	74
Mouth rinse/gargles	23	100
Antimicrobials	23	100
Analgesics/antipyretics	23	100
Antiacid drugs (gastric mucosa protection)	23	100
Respiratory support (O_2_ supplementation)	13	57
Antivirals	7	30
Corticosteroids	6	26
Vitamin supplements	4	17

**Table 7:** Summary of adverse effects from the use of hydrogen peroxide.

ID	Gender and age	Date and time	Route of administration	Adverse effect	Severity of adverse effect^1^	Possible cause (causality)	Security problem^2^	Countermeasure	Consequence of the event^3^
01	Male, 38	05/12/20 20:00 (day 2)	Inhalatory (nebulization)	Momentary shortness of breath	Mild	Accidental use of undiluted solution: using 10 drops undiluted for 3 minutes	Related	Only observation. The discomfort was mild and resolved after 10 minutes	Recovered without after-effects
		05/14/20 19:01 (day 4)	Oral	Nausea	Mild	During the third meal of the day, he felt nauseous when ingesting the treatment quickly and continuously	Related	Only observation. The effect was momentary and disappeared after five minutes	Recovered without after-effects

02	Male, 40	05/16/20 18:00 (day 6)	Inhalatory (nebulization)	Chest tightness	Mild	He had eaten in a hurry, just before doing the nebulization, so indigestion is suspected	Not related	Omeprazole 20 mg, a single dose. The effect disappeared after around 20 minutes	Recovered without after-effects

04	Male, 35	05/22/20 16:00 (day 3)	Oral	Nausea and reflux	Mild	Gastroesophageal reflux disease (chronic) with poor adherence to treatment	Not related	Esomeprazole 40 mg single dose per day, irritant-free diet, and avoiding prolonged fasts. Abandoning oral treatment of his own accord (day 5)	Recovered without after-effects
		05/24/20 19:00 (day 5)	Inhalatory (nebulization)	Palpitations (heartbeat)	Mild	Possible mild intolerance due to gastroesophageal reflux	Not related	Only observation. The effect was momentary and disappeared five minutes later	Recovered without after-effects

05	Male, 19	06/07/20 16:50 (day 1)	Inhalatory (nebulization)	Headache	Mild	He had headache, asthenia, adynamia, chills, and diaphoresis before starting treatment	Not related	Acetaminophen 500 mg, a single dose. Headache disappeared 1 hour later	Recovered without after-effects

06	Female, 21	06/07/20 23:00 (day 1)	Inhalatory (nebulization)	Mild headache and eye irritation	Mild	When nebulizing, the steam escaped through the upper holes of the mask, causing the peroxide contact with the eyes	Related	Nebulization was stopped immediately, and mask openings were canceled	Recovered without after-effects
		06/07/20 23:00 (day 1)	Oral	Nausea	Mild	Drug gastritis (taking 6 drugs without gastric protection)	Not related	Delivery of medications was organized throughout the day, and omeprazole 40 mg was added in a single dose upon waking	Recovered without after-effects

09	Male, 40	06/15/21 18:00 (day 1)	Oral	Odynophagia	Mild	Presence of prior ulcers in pharynx from upper respiratory infection	Not related	Sublingual ketorolac 30 mg, single dose 20 minutes before peroxide	Recovered without after-effects

24	Male, 23	07/02/21 19:00 (day 2)	Oral	Sickness	Mild	Drug gastritis (taking 6 drugs without gastric protection)	Not related	The dosage of drugs is organized throughout the day and omeprazole 20 mg every 12 hours	Recovered without after-effects
		07/02/21 20:00 (day 2)	Inhalatory (nebulization)	Nasal irritation	Mild	Use of salbutamol and fluticasone (nebulized) 10 minutes before use of peroxide	Not related	Use of salbutamol and fluticasone 1 hour before nebulization with peroxide	Recovered without after-effects
32	Male, 30	07/09/21 20:00 (day 6)	Oral	Dizziness and bitter taste	Mild	Peroxide intolerance is suspected because adverse effect appears almost immediately	Related	Reduction of frequency of peroxide to two doses per day	Recovered without after-effects

^1^In accordance with “NORMA Official Mexicana NOM-220-SSA1-2016” (about installation and operation of pharmacovigilance); ^2^in accordance with “Guía de Farmacovigilancia en Investigación Clínica” (COFEPRIS, 2020); ^3^in accordance with “Instructivo de llenado del formato Aviso de Sospechas de Reacciones Adversas de Medicamentos” (COFEPRIS, 2017).

## Data Availability

Data are not available for patient privacy and confidentiality motives.
